# Supplementary material for: Amyloid plaques and normal ageing have differential effects on microglial Ca2+ activity in the mouse brain
Source: Pflugers Arch. 2023 Nov 15;476(2):257–70. doi: 10.1007/s00424-023-02871-3 (PMC10791787; doi:10.1007/s00424-023-02871-3)
Supplement: Supplementary file 1 — Supplementary file1 (DOCX 448 KB) [file 424_2023_2871_MOESM1_ESM.docx]

# Supplementary material for

# Amyloid plaques and normal ageing have differential effects on microglial Ca^2+^ activity in the mouse brain

Pablo Izquierdo,^1^ Renaud B. Jolivet,^1,2^ David Attwell,^1,*^ Christian Madry,^1,3,*^

^1^Department of Neuroscience, Physiology and Pharmacology, University College London, London, WC1E 6BT, UK.

^2^Current address: Maastricht Centre for Systems Biology (MaCSBio), Maastricht University, Paul-Henri Spaaklaan 1, 6229 EN Maastricht, The Netherlands.

^3^Current address: Charité – Universitätsmedizin Berlin, Corporate member of Freie Universität Berlin and Humboldt Universität zu Berlin, Institute of Neurophysiology, 10117 Berlin, Germany.

*Correspondence: [christian.madry@charite.de](mailto:christian.madry@charite.de) or [d.attwell@ucl.ac.uk](mailto:d.attwell@ucl.ac.uk)

## Supplementary Figure 1. Proximity to Aβ plaques increases the lysosomal burden in microglia from *App*^NL‑G-F^ mice.


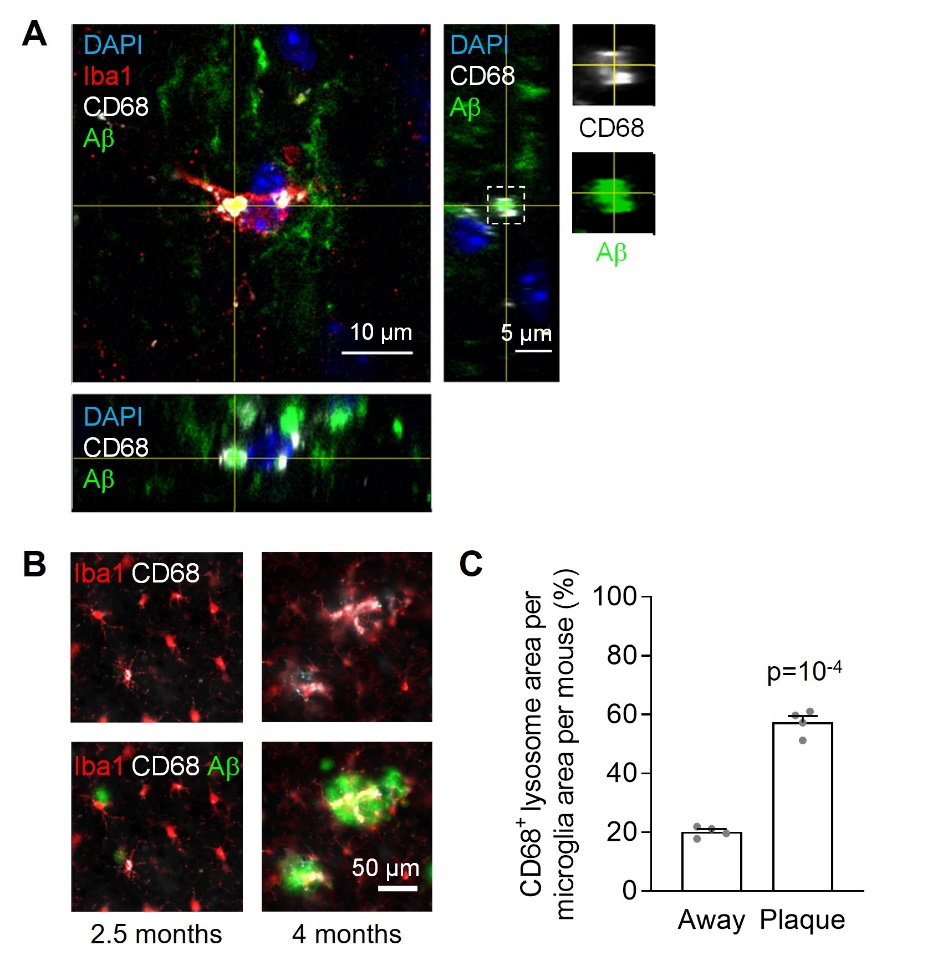


**A)** Microglial cell with phagocytosed Aβ in a 4‑month-old *App*^NL-G-F^ mouse. Orthogonal projections at the level of the crosshairs show internalisation of Aβ by the microglial cell (intensity of Aβ channel is adjusted to see internalised Aβ more clearly). Right panels show the indicated area (white dashed square) at higher magnification to highlight the presence of Aβ within the CD68^+^ lysosome. **B)** Representative images of hippocampal microglia (Iba1, red) in *App*^NL-G-F^ mice at 2.5 months (left) and 4 months of age (right). At 4 months, when large Aβ plaques (green) have already developed, microglia show increased expression of the lysosomal marker CD68 (white) as they cluster around them. **C)** Quantification of the fraction of the microglial area covered by CD68 in the hippocampus of four 4-month-old *App*^NL-G-F^ mice, showing a sharp increase in plaque microglia compared to cells away from plaques.

## Supplementary Figure 2. Example of Hermite polynomial subtraction of baseline drift for GCaMP5g signal.


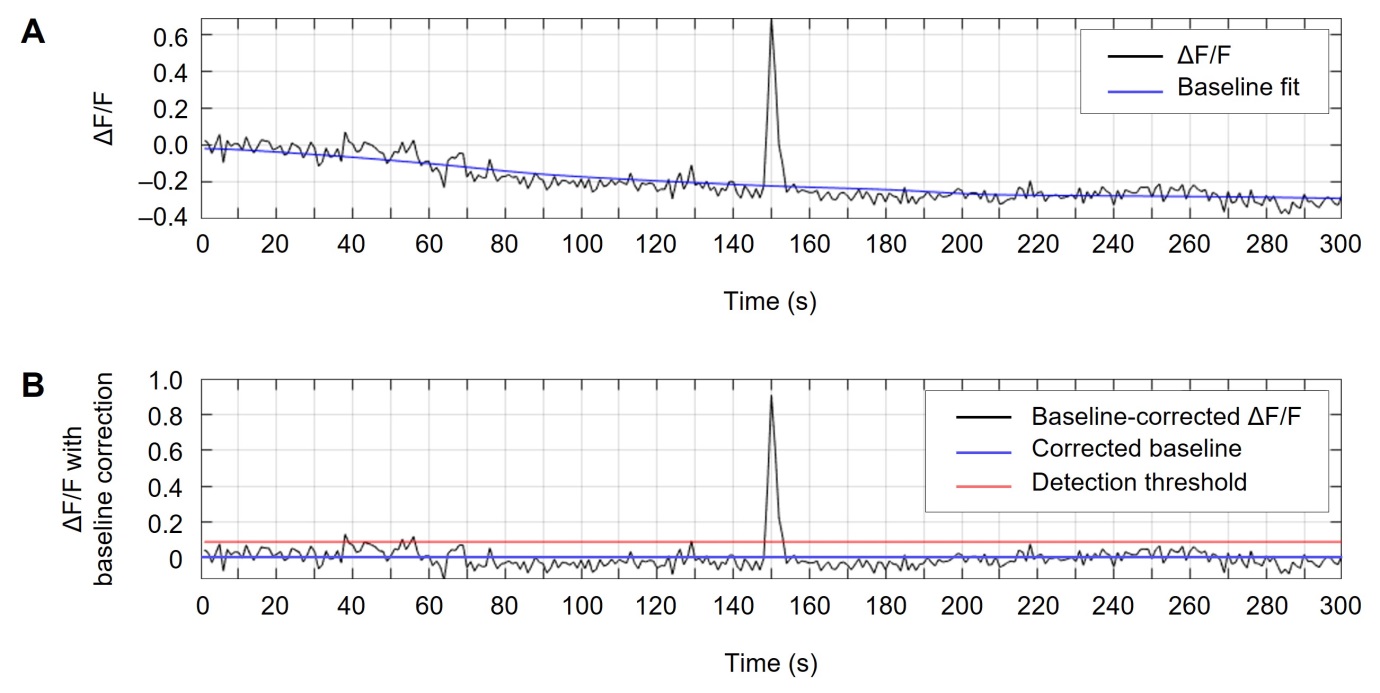


**A**) Raw fluorescence intensity for GCaMP5g (ΔF/F), with a Hermite polynomial baseline fit shown (blue line, see Materials and Methods). **B**) Adjusted trace with the fitted baseline in (A) subtracted, a blue line shown at zero and a red line showing the detection threshold defining whether transients occur (calculated as 2.25 standard deviations from the baseline).

## Supplementary Figure 3. Amplitude of calcium transients was not significantly different between microglia from wild-type and *App*^NL-G-F^ mice.

**
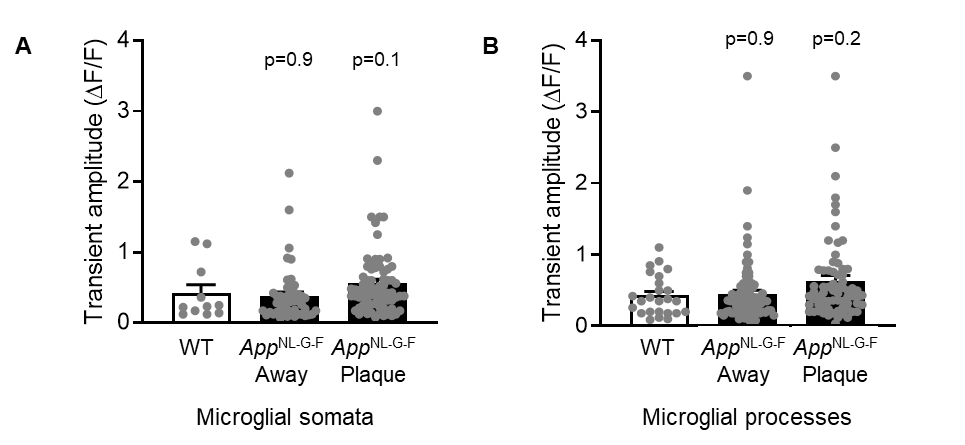
**

**A)** Amplitude of calcium transients in microglial somata from *App* wild-type (WT) and
*App*^NL-G-F^ mice at, or >50 µm away from, Aβ plaques. **B)** As (A), but for processes.

Supplementary Figure 4. Amplitude of calcium transients was not significantly different between microglia from young adult and old mice.

**
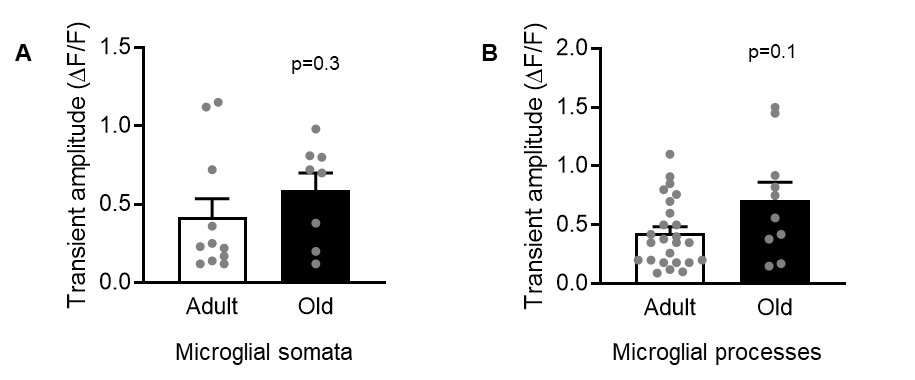
**

**A)** Amplitude of calcium transients in microglial somata from P120–130 (young adult) and P300–310 (old) mice. **B)** As (A), but for processes.
